# Genetic characterization of Lassa virus strains isolated from 2012 to 2016 in southeastern Nigeria

**DOI:** 10.1371/journal.pntd.0006971

**Published:** 2018-11-30

**Authors:** Olamide K. Oloniniyi, Uche S. Unigwe, Sayaka Okada, Mayuko Kimura, Shota Koyano, Yukiko Miyazaki, Michael O. Iroezindu, Nnenna A. Ajayi, Chinedu M. Chukwubike, Nneka M. Chika-Igwenyi, Anne C. Ndu, Damian U. Nwidi, Haruka Abe, Shuzo Urata, Yohei Kurosaki, Jiro Yasuda

**Affiliations:** 1 Department of Emerging Infectious Diseases, Institute of Tropical Medicine (NEKKEN), Nagasaki University, Nagasaki, Japan; 2 Graduate School of Biomedical Sciences and Program for Nurturing Global Leaders in Tropical and Emerging Communicable Diseases, Nagasaki University, Nagasaki, Japan; 3 Department of Medicine, Federal Teaching Hospital Abakaliki, Abakaliki, Ebonyi, Nigeria; 4 Department of Medicine, University of Nigeria Teaching Hospital, Ituku-Ozalla, Enugu, Nigeria; 5 Department of Medicine, College of Medicine, University of Nigeria, Ituku-Ozalla, Enugu, Nigeria; 6 Department of Microbiology, University of Nigeria Teaching Hospital, Ituku-Ozalla, Enugu, Nigeria; 7 Department of Community Medicine, College of Medicine, University of Nigeria, Ituku-Ozalla, Enugu, Nigeria; 8 National Research Center for the Control and Prevention of Infectious Diseases (CCPID), Nagasaki University, Nagasaki, Japan; The University of Kansas, UNITED STATES

## Abstract

Lassa virus (LASV) is endemic in parts of West Africa where it causes Lassa fever (LF), a viral hemorrhagic fever with frequent fatal outcomes. The diverse LASV strains are grouped into six major lineages based on the geographical location of the isolated strains. In this study, we have focused on the lineage II strains from southern Nigeria. We determined the viral sequences from positive cases of LF reported at tertiary hospitals in Ebonyi and Enugu between 2012 and 2016. Reverse transcription-polymerase chain reaction (RT-PCR) showed that 29 out of 123 suspected cases were positive for the virus among which 11 viral gene sequences were determined. Phylogenetic analysis of the complete coding sequences of the four viral proteins revealed that lineage II strains are broadly divided into two genetic clades that diverged from a common ancestor 195 years ago. One clade, consisting of strains from Ebonyi and Enugu, was more conserved than the other from Irrua, although the four viral proteins were evolving at similar rates in both clades. These results suggested that the viruses of these clades have been distinctively evolving in geographically separate parts of southern Nigeria. Furthermore, the epidemiological data of the 2014 outbreak highlighted the role of human-to-human transmission in this outbreak, which was supported by phylogenetic analysis showing that 13 of the 16 sequences clustered together. These results provide new insights into the evolution of LASV in southern Nigeria and have important implications for vaccine development, diagnostic assay design, and LF outbreak management.

## Author summary

Lassa fever (LF) is a viral hemorrhagic fever caused by Lassa virus (LASV). The different LASV strains are grouped into lineages based on the geographical location of the isolated strains. The aim of our study was to characterize the lineage II strains in southern Nigeria. We sequenced LASV RNA genome from positive cases of LF between 2012 and 2016 which were reported at tertiary hospitals in Ebonyi and Enugu in southeastern Nigeria. Phylogenetic analysis of the viral proteins showed the division of lineage II strains into two genetic clades with one clade being more conserved than the other despite evolving at similar rates. Also, our phylogenetic analysis supported the role of human to human transmission in the 2014 outbreak, in keeping with the epidemiological data. These results provide additional information on the evolution of LASV in southern Nigeria and LF outbreak management.

## Introduction

Lassa virus (LASV) is the causative agent of Lassa fever (LF), a viral hemorrhagic fever with manifestations that range from asymptomatic to an acute, severe form associated with significant mortality (up to 50% in hospitalized patients) [1, 2]. In symptomatic cases, the initial manifestations are usually those of a generalized flu-like symptoms which renders clinical diagnosis difficult owing to several possible differential diagnoses [3–5]. LF is associated with significant maternal morbidity during pregnancy, with a range of clinical features that include vaginal bleeding, threatened abortion, puerperal infection, intrauterine fetal death, premature labor, and eventual mortality, all of which depend on the stage of the pregnancy [6]. LF is endemic in West Africa, where it is estimated to affect up to 2 million people annually [7]. Furthermore, owing to the prevalence of global travel, the occurrence of LF cases in non-endemic regions is an emerging matter of concern [8, 9]. LASV is primarily transmitted via contact with infected tissue, blood, or excreta of *Mastomys natalensis*, which is the reservoir host for the virus, although it has also been recently isolated from other small rodents such as *Hylomyscus pamfi* and *Mastomys erythroleucus* [10, 11]. Human-to-human transmission is also one of the routes of transmission, especially in hospital settings, and this has been estimated to be responsible for 20% of LASV transmissions although a recent study demonstrated human to human transmission in 3% of 169 LASV sequences that were analyzed [12–16].

**Fig 1:**
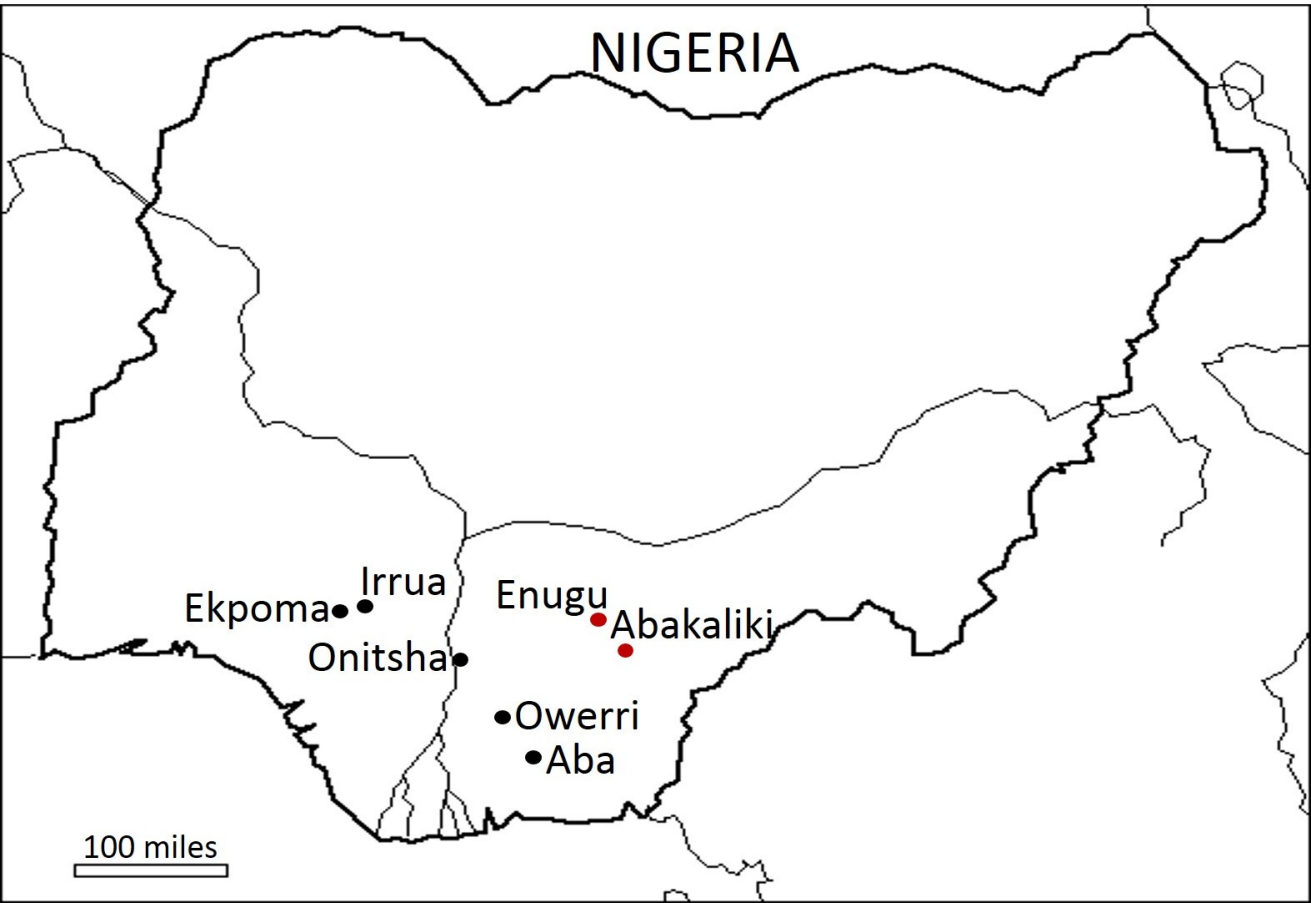
Map of Nigeria showing the sites from where lineage II strains were isolated. Sites discussed in this study are highlighted in red.

LASV is an enveloped, bi-segmented RNA virus of family *Arenaviridae* and genus *Mammarenavirus*, which consists of different species of arenaviruses that infect mammals. Historically, *Mammarenavirus* has been divided into the New World and Old World groups based on serological properties. LASV belongs to the Old World arenaviruses, and its RNA genome contains large (L) and small (S) segments that encode two proteins each with an ambisense strategy. The matrix RING finger protein (Z) and RNA-dependent RNA polymerase (L) are encoded by the L segment, whereas the nucleoprotein (NP) and glycoprotein precursor (GPC) are encoded by the S segment [17]. Phylogenetic analyses have shown that LASV nucleotide sequences cluster into six lineages (I, II, III, IV, V, and VI) based on geographical locations [18–20]. Lineage I, which contains the first strain of LASV identified in 1969, occurs in northeastern Nigeria; lineage II strains were isolated from Irrua, Ekpoma, Onitsha, Abakaliki, Aba, and Owerri in southeastern and south-central Nigeria (Fig 1). However, outbreaks of LF in Nigeria are being reported since January 2018, with cases being detected in other parts of southern Nigeria with no previous report of LASV infection [21]. Lineage III strains were isolated from north-central Nigeria, whereas lineage IV strains were isolated from other Mano River Union countries of Sierra Leone, Guinea, and Liberia [18, 22–25]. Recent studies have also suggested the existence of a fifth lineage (V), which consists of sequences isolated from other West African countries such as Mali, Cote d’Ivoire, and Ghana [19]. Furthermore, the virus isolated from a cluster of human infections in Togo in 2016 represented a new lineage (VI) of LASV [20].

The delineation of LASV into distinct lineages reflects its diversity. Sequence diversity is higher (as high as 24.6%) between lineages, whereas they are more conserved (9.6%) within each lineage [14, 18, 22]. This inter-lineage heterogeneity has practical implications for developing vaccines and effective diagnostic assays, especially nucleic acid detection tests such as reverse transcription polymerase chain reaction (RT-PCR). RT-PCR could not detect different LASV lineages with high sensitivity [26]. Of the complete LASV lineage II sequences available in GenBank, a significant proportion was isolated mainly from Irrua, and one out of > 90 sequences was isolated from Abakaliki, and none from Enugu. However, the isolation of new sequences from different areas will lead to better characterization of LASV diversity both within and between lineages, which will be important for the prevention and control of this disease; in addition, it will provide new insights into the pattern of LASV dissemination and evolution [24, 25].

In this study, we identified LASV-infected cases from among patients with suspected LF who were admitted in two teaching hospitals in Enugu and Abakaliki in southeastern Nigeria from 2012 to 2016. To investigate the molecular epidemiology of LASV in southeastern Nigeria, we determined 11 nearly-complete genome sequences of the viruses from these patients. Phylogenetic analysis of all four genes showed that lineage II sequences were broadly divided into clades IIA and IIB. Clade IIA sequences are more conserved than clade IIB sequences. We also confirmed the suspected role of human-to-human transmission in the 2014 outbreak using clinical epidemiological data and phylogenetic analysis of the partial sequences from positive samples.

## Materials and methods

### Ethics statement

This study was approved by the Institutional Ethical Committee of Nagasaki University (approval number 140829131) and the ethical committee on research of the Enugu state Ministry of Health (approval number MH/MSD/EC/0129). Samples were collected as part of the response to contain the outbreak, and informed consent was obtained from all patients, all of whom were adults, in accordance with the protocols of the Federal Teaching Hospital Abakaliki (FETHA) and University of Nigeria Teaching Hospital (UNTH), Enugu.

### Patients and sample collection

Blood samples were collected from patients with suspected acute LF, who were admitted to the UNTH, Enugu, and FETHA from 2012 to 2016. A diagnosis of acute LF was made based on clinical signs and symptoms of fever, headache, and/or hemorrhage, contact history with a suspected or confirmed LF patient, as well as the discretion of the managing physician. All patients were managed based on the standard protocol available at both centers for management of such cases. Sera from the collected blood samples were inactivated using Trizol LS reagent (Life Technologies, Carlsbad, CA, USA) and then shipped to Nagasaki University following the approved protocol.

### RT-PCR

RNA was extracted from inactivated serum samples after phase separation with 200 μl chloroform using the QIAmp viral RNA mini kit (Qiagen, Hilden, Germany) according to the manufacturer’s protocol. LASV was detected using RT-PCR and 36E2 and LVS-339-rev primer sets according to a previously published protocol [26].

### Viral gene sequencing

The L and S segments of the LASV genome were sequenced using the diagnostic RT-PCR assay from LASV-positive samples. The sequences of the L and S segments contained the complete coding sequences of the respective genes on each segment, as well as the intergenic regions. However, the terminal 5′ and 3′ regions were not determined. The segments were mostly sequenced using new overlapping primers (S1 and S2 Tables) modeled on LASV strain Nig08-04 (GenBank accession numbers GU481068 and GU481069) sequence. The 7-kb L segment was amplified using primers that produced three 2-kb amplicons and one 1-kb amplicon, whereas the 3.5-kb S segment was amplified with primers that resulted in four 0.9-kb amplicons. RT-PCR was performed using PrimeScript II High Fidelity one step RT-PCR kit (Takara Bio, Shiga, Japan) and 2 μl extracted viral RNA in a reaction volume of 25 μl according to the manufacturer’s instruction. RT-PCR was performed at 45°C for 10 min and 94°C for 2 min, followed by 45 cycles of 98°C for 10 s, 55°C for 15 s, and 68°C for 10 or 25 s (depending on the size of the amplicon), and a final extension at 68°C for 6 min. The RT-PCR products were verified via agarose gel electrophoresis and purified using the QIAquick gel extraction kit (Qiagen, Hilden, Germany). The purified products were subsequently sequenced using the BigDye Terminator v1.1 sequencing kit (Life Technologies, Carlsbad, CA) according to the manufacturer’s instructions with the aforementioned overlapping primers. For the L segment amplicons, additional sequencing primers were designed to enable complete coverage of the large amplicons (S2 Table). For the LASV-positive samples that could not be sequenced using the new primer sets, the RT-PCR amplicons were purified and sequenced using the 36E2 and LVS-339-rev primer set. All sequences were analyzed using the 3130xl Genetic Analyzer (Applied Biosystems) and assembled using the Genetyx v.11 software (Genetyx, Tokyo, Japan).

### Phylogenetic analysis

Available full-length sequences for the L and S segments of LASV in GenBank were imported into MEGA v7.0.21 and aligned with the new LASV sequences using ClustalW. The resulting alignments were trimmed to include only the viral protein-coding regions for each of the LASV genes. Model testing was also performed in MEGA to determine the best model for analyzing each of the aligned gene sequences. A Tamura-Nei model with gamma distributed and invariant sites (TN93, G+I) was used for Z protein-encoding gene (henceforth referred to as Z gene), Tamura-3 parameter with gamma distributed sites (T92, G) for NP gene, and General Time reversible mode with gamma distributed and invariant sites (GTR, G+I) for both L and GPC genes. Phylogenetic and molecular evolutionary analyses were performed using the above-mentioned models with the Bayesian Markov chain Monte Carlo (MCMC) method via BEAST v1.8.4. The analyses were performed under an uncorrelated lognormal relaxed molecular clock with a constant population size. The MCMC methods were performed for 165–250 million steps (depending on the size of the gene) for all analysis to achieve convergence and an effective sample size of 200 or higher. The time-scaled Maximum Clade Credibility (MCC) trees were generated using Tree Annotator v1.8.4 after removing the first 10% trees as burn in. The phylogenetic tree was generated using maximum likelihood (ML) method with 1,000 bootstrap replications using MEGA V7.0.21. The ML tree was rooted with the Mopeia virus (accession no. JN561684.1) as the outgroup.

## Results

### Lineage II comprises of two clades

We identified LASV infections in 123 patients suspected to have LF in Abakaliki and Enugu from 2012 to 2016, and detected the virus genome in 29 patients (23.6%) by conventional RT-PCR. We successfully sequenced 11 nearly-complete L and S segments, in addition to one GPC sequence, from Abakaliki (Table 1). Most of the complete lineage II sequences available on GenBank were obtained from patients living in or around Irrua, where the Irrua Specialist hospital (ISTH) is located, and the sequenced samples were collected between 2008 and 2013 [14]. We also obtained 17 partial sequences from the diagnostic RT-PCR amplicons of Abakaliki samples, with the exception of one sequence from Enugu (S3 Table).

**Table 1:** Data summary.

	Year of collection			
	2012	2013	2014	2016
Suspected cases	56	10	41	16
RT-PCR confirmed	4	3	16	6
Sequenced				
S segment				
GPC	1	1	5	5
NP	1	1	5	4
L segment				
L	1	1	5	4
Z	1	1	5	4

**Fig 2:**
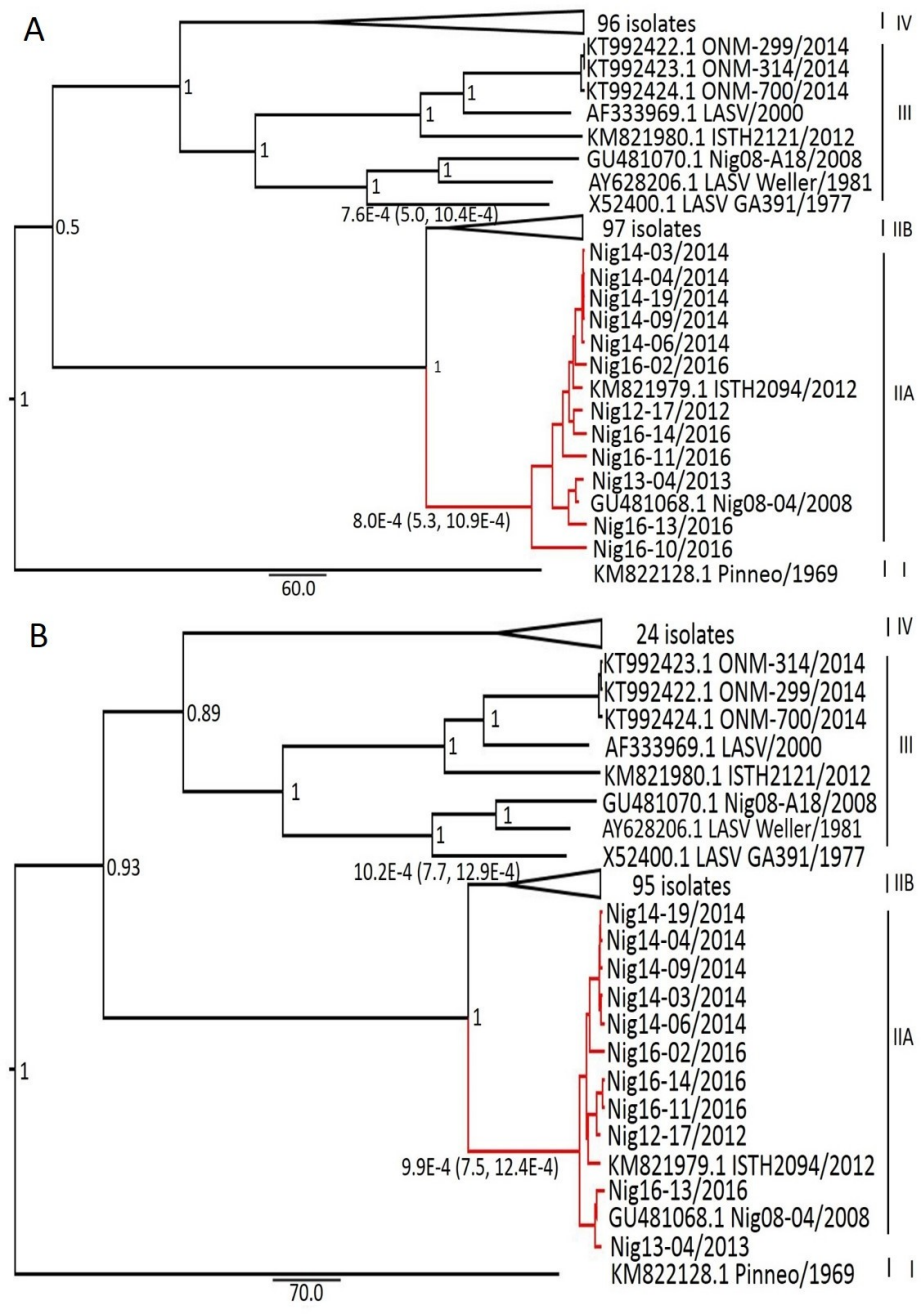
Maximum clade credibility (MCC) trees for GPC and NP genes. Complete nucleotide coding sequences of the GPC (A) and NP genes (B) were aligned, and phylogenies were inferred using the Bayesian Markov chain Monte Carlo method with the following parameters: General time reversible model plus gamma distributed with Invariant sites (GTR G+I) for the GPC gene and Tamura 3-parameter plus gamma distributed (T92+G), relaxed lognormal clock for NP gene. Posterior support was provided at the nodes. Branches to lineage IIA sequences are highlighted in red. Sequences in lineage IIB and IV have been compressed for clarity.

**Fig 3:**
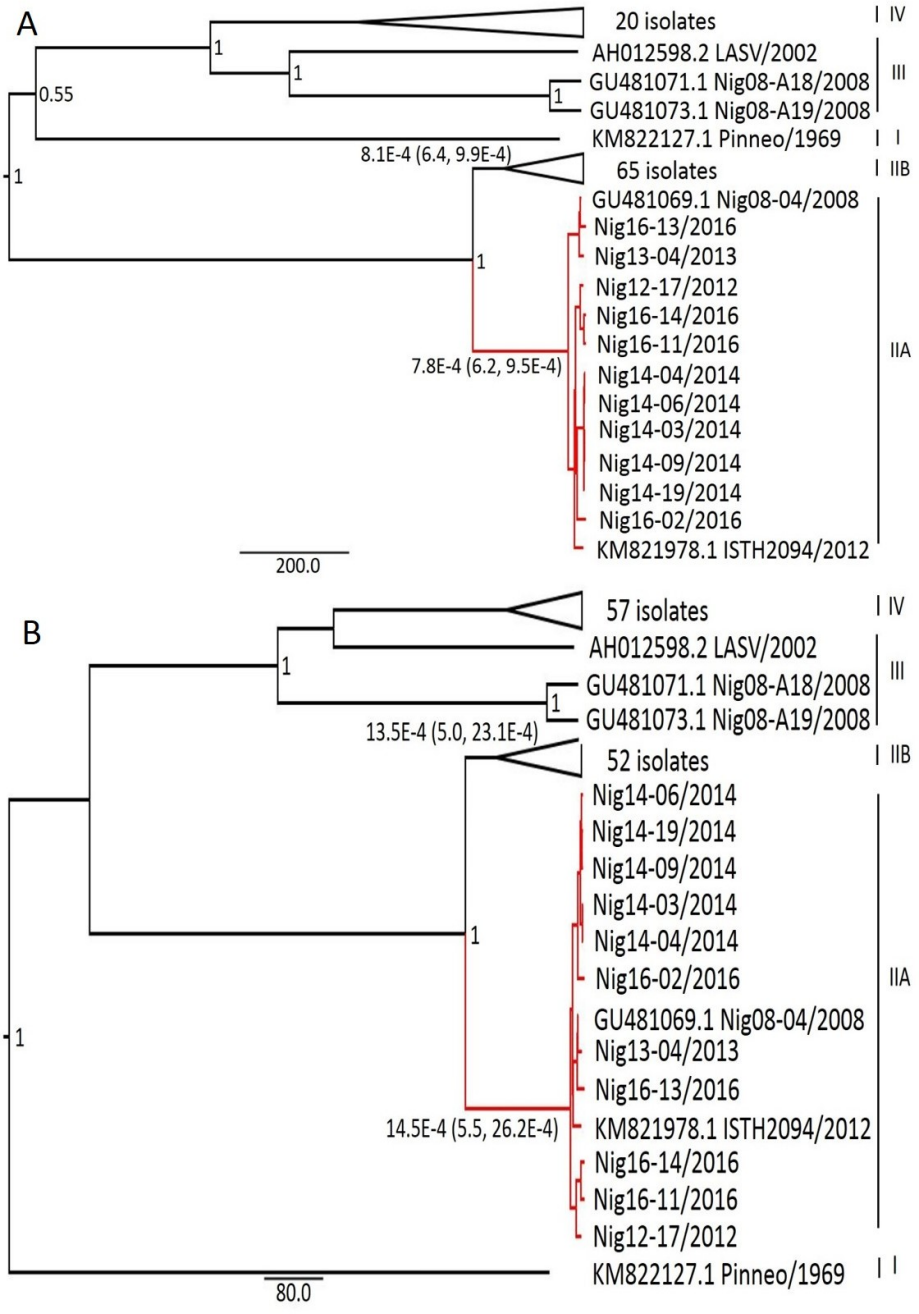
Maximum clade credibility (MCC) trees for L and Z protein genes. Complete nucleotide coding sequences of L (A) and Z protein genes (B) were aligned and phylogenies were inferred using the Bayesian Markov chain Monte Carlo method with the following parameters: General time reversible model plus gamma distributed with invariant sites (GTR+G+I) for the L gene, Tamura-Nei model plus gamma distributed with invariant sites (TN93+G+I) for the Z gene using relaxed lognormal clock for both genes. Posterior supports were provided at the nodes. Branches to lineage IIA are highlighted in red. Sequences in lineage IIB and IV have been compressed for clarity.

Time-scaled phylogenetic tree and the evolutionary rates of the GPC, NP, L, and Z genes were analyzed using our novel sequences, together with other complete LASV sequences present in GenBank, following the Bayesian MCMC method (Figs 2 and 3). Across all the proteins encoded by L and S segments, the topology of the MCC tree revealed the presence of four lineages with posterior probabilities of 0.7 or higher for each major node in all the four viral protein MCC trees, which were consistent with the results of previous studies [14, 18, 22]. The overall topologies of the trees have lineage I sequences as the basal sequence, with the exception of the L gene tree, in which lineage II sequences were basal, which is indicative of a recombination event in the L segment. In particular, our sequences clustered in lineage II, which was expected based on the geographical origin of our samples. However, a clear demarcation of lineage II sequences into two distinct major clades was evident from our analysis; the southeast Nigeria clade (clade A) contained all the new sequences in addition to two other sequences (LASV Nig08-04 and ISTH2094-NIG/2012), whereas the south-central Nigeria clade (clade B) contained sequences isolated in Irrua, Ekpoma, and Onitsha. This demarcation into clades A and B was present in the tree of all the genes and was well supported with posterior probabilities of 1 (Figs 2 and 3). Furthermore, all clade A sequences were from the eastern part of southern Nigeria except that of ISTH2094-NIG/2012, which was isolated in Irrua (the lineage II strains were from this place), whereas clade B sequences were from the western part. The most recent common ancestor (tMRCA) from which both the clades diverged existed approximately 136–195 years ago (Table 2). The evolutionary rate of clade A and clade B strains, respectively, across all the genes were 8.0 and 7.6 × 10^−4^ substitutions site^-1^ year^-1^ for GPC, 9.9 and 10.2 × 10^−4^ substitutions site^-1^ year^-1^ for NP, 7.8 and 8.1 × 10^−4^ substitutions site^-1^ year^-1^ for L, and 14.5 and 13.5 × 10^−4^ substitutions site^-1^ year^-1^ for the Z gene (Table 2). These results suggest that clade A and B strains are evolving at similar rates.

**Table 2:** Evolutionary rate and year of divergence for each Lassa virus gene.

		Age (yr) of MRCA before present				
Gene	Mean rate (95% HPD)^1^	Lineage I	II	III	IV	IIA/B
GPC	7.7 (6.2–9.3)	605 (465–758)	564 (444–697)	429 (340–562)	289 (226–360)	170 (132–209)
NP	10.1 (8.2–12.2)	615 (473–766)	522 (409–641)	439 (348–538)	112 (88–137)	142 (112–173)
L^2^	8.4 (7.2–9.6)	965 (817–1124)	915 (779–1066)	630 (531–732)	382 (324–443)	195 (165–226)
Z	16.9 (8.6–25.9)	767 (343–1294)	567 (264–984)	335 (134–575)	103 (52–163)	136 (56–231)

^1^Mean rate × 10^−4^ substitutions site^-1^ year^-1^

^2^ Lineage I and II are inverted.

MRCA, Most recent common ancestor.

### Sequence diversity of the clades in LASV lineage II

Next, we analyzed sequence diversity at nucleotide and amino acid levels within and between lineage II sequences that were used for phylogenetic analysis. The overall diversity between the sequences was highest within the Z gene and lowest in the NP gene (Table 3). Both Z and L genes were more diverse than the NP and GPC genes overall, although this might partially be because of less sequences of L segment genes than the S segment genes in GenBank. Clade A sequences were more conserved than clade B sequences, with values ranging from 2.5 to 3.6% compared to 5.8–8.2% in clade B at the nucleotide level, and 0.9–3.6% compared to1.2–6.4% in clade B at the amino acid level across all genes (Table 3). The lower diversity within clade A sequences than in clade B was maintained across all genes at the nucleotide and amino acid levels, with the exception of the GPC gene. Even when the 2014 sequences and other sequences in clade A were analyzed separately because of the high sequence identity between the 2014 samples (Table 3), the pattern of the clade A sequences were more conserved than those of clade B sequences (with the exception of GPC) based on differences in nucleotide and amino acid composition.

**Table 3:** Differences in sequence within lineage II strains.

	**Percentage of nucleotide difference**			
	**GPC**	**NP**	**L**	**Z**
Lineage II	8 (0–16.0)	7.7 (0–16.2)	9.5 (0–18.3)	11.2 (0–21.0)
Clade IIA	3.6 (0.1–7.5)	2.9 (0.2–4.9)	2.8 (0–5.1)	2.5 (0–5.0)
2014	0.2 (0.1–0.3)	0.3 (0.1–0.5)	0.1 (0.1–0.2)	0.1 (0–0.3)
Other	4.3 (1.2–7.5)	3.1 (0.4–4.9)	3.4 (0.6–5.1)	3.3 (0.7–5.0)
Clade IIB	6.2 (0–14.5)	5.8 (0–12.9)	6.5 (0–15.1)	8.2 (0–20.7)
	**Percentage of amino acid difference**			
	**GPC**	**NP**	**L**	**Z**
Lineage II	2.0 (0–5.7)	1.5 (0–5.5)	4.8 (0–9.7)	9.1 (0–20.2)
Clade IIA	1.7 (0–2.9)	0.9 (0–3.2)	1.7 (0.1–3.1)	3.6 (0–7.1)
2014	0.3 (0–0.8)	0.5 (0.2–1.1)	0.2 (0–0.4)	0.4 (0–1.0)
Other	2.0 (0.8–2.9)	1.0 (0–3.2)	1.9 (0.4–2.8)	4.1 (0–7.1)
Clade IIB	1.6 (0–4.7)	1.2 (0–3.0)	3.1 (0–7.9)	6.4 (0–13.1)

### Role in human-to-human transmission during the 2014 outbreak

**Fig 4:**
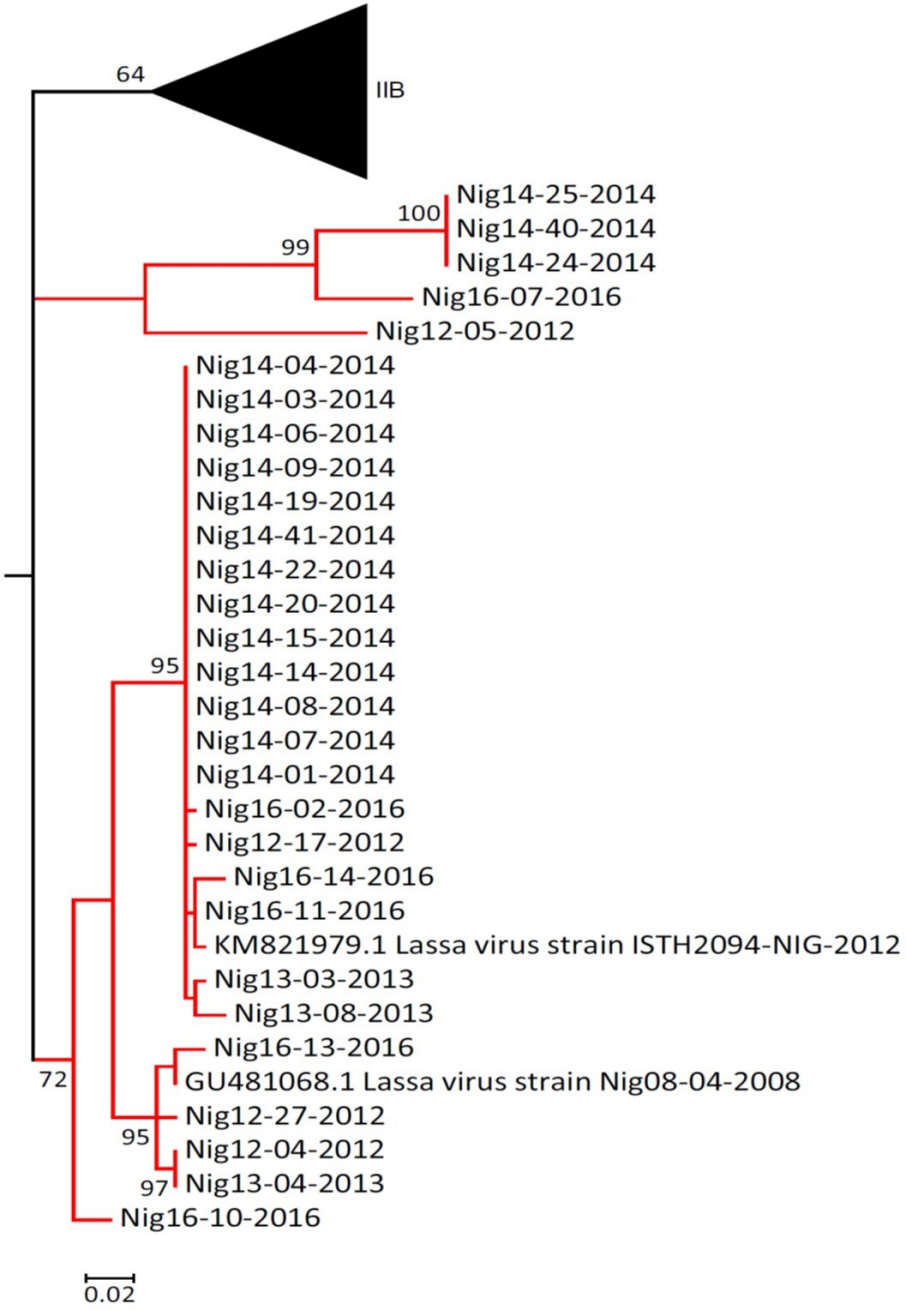
Maximum likelihood subtrees showing lineage II sequences. Partial sequences from diagnostic RT-PCR amplicons of confirmed cases of Lassa virus between 2012 and 2016 were aligned with other available sequences from GenBank. The length of the aligned sequences was 231 bp starting from the GPC start codon. Gaps or missing sequences were treated by complete deletion in the analysis. Phylogeny was inferred after 1,000 bootstrap replications, and bootstrap support of 50 and above are shown at nodes. The tree was rooted with Mopeia virus (not shown).

In 2014, a putative transmission chain for cases of LF was observed in FETHA. The suspected index case was a woman in the late third trimester of pregnancy, who presented with vaginal bleeding and lower abdominal pain, and was managed as a case of placenta abruption. She had an emergency cesarean section and delivered a live male neonate. She underwent a second surgery because of bleeding at the operation site, but both mother and baby died eventually. Subsequently, there was an outbreak of LF among the health workers who were involved in the management of the mother and child. To confirm the epidemiological link in these cases, partial sequences derived from the diagnostic RT-PCR amplicons were used for analysis. Thirteen of the 2014 sequences clustered together, whereas three clustered together with a bootstrap support of 95 and 100, respectively (Fig 4). All 13 sequences that clustered together were from individuals who had primary or secondary contact with the index case. Additionally, high sequence identity (98.9 to 100%) was observed among these 13 sequences, which supported the transmission links between these cases.

## Discussion

In this study, we examined in detail the molecular epidemiology of LASV in southeastern Nigeria by sequencing the LASV genome from separate sites in the region. In the topology of the MCC trees, lineage I was basal, except for the L gene where lineage II sequences were basal. This finding was similar to the results of a previous study in which lineage II sequences were also basal in the L gene MCC tree [19]; however, combination with the topology of the Z gene, where lineage I was basal, indicated the possibility of recombination between the L and Z genes. Furthermore, when the MCC tree for the Z gene was drawn using the same model (GTR, G+I, figure not shown) as the L gene, the topology of the tree was identical to that of the tree drawn with TN93, G+I. A previous study by Ehichioya et al. (2011) also showed a difference in the positioning of the basal sequence in the phylogenetic analysis of GPC and NP genes, although no evidence of recombination was obtained [22]. Previous studies on the molecular epidemiology of LASV have hinted at the existence of clades within lineage II strains from southeastern Nigeria [14, 18, 22]. However, using our new sequences, we have conclusively demonstrated that lineage II strains can be broadly divided into two clades. While previous studies have demonstrated this using the NP, GPC, and L genes [18, 22], we successfully showed this also on the Z gene. The MCC tree for the Z gene clearly showed the existence of four lineages.

The number of viral genome sequences determined in south-central Nigeria is increasing because of intensive international collaborative research in Irrua, which has revealed the evolutionary history of the virus in Nigeria. However, the sequences available in public sequence database for our region of interest in southeastern Nigeria are limited. Unbiased viral genome information from different areas will augment our understanding of the epidemiology and evolution of this highly diverse virus. Previous studies have shown geographical clustering of LASV sequences over large geographical distances, as well as over relatively short distances within Sierra Leone [18, 24]. Similarly, we observed a pattern of LASV clustering based on geographical location in southern Nigeria. Furthermore, a previous phylogenetic analysis using partial sequences suggested that lineage II sequences had evolved from Owerri to Irrua/Ekpoma [22]. If the topology of the tree obtained from that analysis were to be combined with our current result, it can possibly be inferred that clade A strains occupy the southeastern part in Abakaliki and Enugu, whereas clade B strains are in the south-central part, including Onitsha, Ekpoma, and Irrua (Fig 1). An obvious caveat is that the precise area occupied by lineage II strains in southern Nigeria is not yet completely delineated, and a study on the diversity of sampling locations will be important in filling this gap.

Furthermore, it is noteworthy that the complete clade A sequences were from Abakaliki, with the exception of ISTH2094-NIG/2012, which was isolated from Irrua. A possible explanation for this might be that the patient from whom the sample was isolated was referred to ISTH from another part of southeastern Nigeria. Human mobility can make identification of the exact location of LASV transmission difficult, which has been observed in previous studies [24]. Indeed, as ISTH is a reference center for LF management, patients who were referred there for further disease management were also included in our anaysis. Another indication of this was the presence of the sequence LASV ISTH2121 (KM821980) in lineage III clusters of the NP and GPC tree, even though it was isolated from Irrua. A better method may involve labeling sequences based on the location of the patient prior to or around the time they developed symptoms. In addition, the sequence of LASV circulating in *Mastomys* in Abakaliki or Enugu should also be examined to understand the genetic information of this virus endemic in southeastern Nigeria.

Analysis of all LASV genes, including the Z gene, supports the emergence of LASV in Nigeria > 1,000 years ago, which is consistent with the findings of previous studies [14, 19, 22]. Clade A and B strains appear to have diverged from an ancestral virus of lineage II 195 years ago, following the introduction of LASV into the southeastern region from lineage I [22]. Clade A and B strains occupy separate areas in southern Nigeria, although they are evolving at similar rates. This may be due to the introduction and maintenance of the virus in reservoir hosts, where they evolve independently without interacting with each other. The barrier to the interaction between the reservoir hosts in separate foci could be the river Niger and its tributaries, with clade A strains being on the eastern side of the river, whereas clade B were restricted on the western side (Fig 1). More studies on LASV in reservoir hosts will shed light on this particular dilemma.

Ever since the identification of LASV in Nigeria in 1969, numerous studies have demonstrated the role of human-to-human transmission in LF outbreaks, especially as nosocomial transmission in hospital settings [10, 12, 27–29]. All these studies have relied on case histories and contact tracing; however, in this study, we have successfully supported our epidemiological data using phylogenetic analysis. The clustering of the 2014 sequences together across the various topologies of the different genes of LASV strongly supports the role of human-to-human transmission in this particular outbreak. The use of standard epidemiological data together with phylogenetic analysis can offer important insights regarding control of LF outbreak. The non-specific nature of early symptoms of LF [1, 2], coupled with the absence of readily available diagnostic assays in resource-poor settings, makes the nosocomial dissemination of LF an ever-lingering public health crisis. The development of vaccines and rapid diagnostic kits, together with increased awareness among health workers, might help reduce the incidence of human-to-human transmission of LF.

## Supporting information

S1 Table Primer list for overlapping RT-PCR. (DOCX)

S2 Table Sequencing primers for L segment. (DOCX)

S3 Table Strains isolated in this study. (DOCX)
