# Supplementary material for: Genetic characterization of Lassa virus strains isolated from 2012 to 2016 in southeastern Nigeria
Source: PLoS Negl Trop Dis. 2018 Nov 30;12(11):e0006971. doi: 10.1371/journal.pntd.0006971 (PMC6267959; doi:10.1371/journal.pntd.0006971)
Supplement: S1 Table — (DOCX) [file pntd.0006971.s001.docx]

**S1 Table. Primer list for overlapping RT-PCR.**

| Primers | Sequences (5′→3′) | Position¹ |
| --- | --- | --- |
| *S segment* |  |  |
| 36E2 | accggggatcctaggcattt | 5-24² |
| LASV-seq-1R | cattgccctcagagtcagagagg | 844 - 866 |
| LASV-seq-1.5F | aacggtgtcctacagac | 602 - 618 |
| LASV-seq-1.5R | tctcattacacttcgcaatgg | 939 - 959 |
| LASV-seq-2F | cctattggatacctaggactg | 761 - 781 |
| LASV-seq-2R II | tgtcaggaggactcaatatacc | 1659 - 1680 |
| LASV-seq-2.5F | aagccacacagactcaac | 1442 - 1459 |
| LASV-seq-2.5R | tgtcacatgcacactgga | 1769 - 1786 |
| LASV-seq-3F | caggacgactttgggacttg | 1595 - 1614 |
| LASV-seq-3R | gatgggggtaacatgttggaaac | 2502 - 2524 |
| LASV-seq-3.5F | ggttcttgaagctatgtaggg | 2330 - 2350 |
| LASV-seq-3.5R | aatatggtagataccaagaagagc | 2591 - 2614 |
| LASV-seq-4F | cccagggacctttttacctttag | 2433 - 2455 |
| LASV-seq-4R | cgcacagtggatcctaggc | 3378 - 3396 |
| *L segment* |  |  |
| LASV08-04 L1F III | aagacttgtcttgtgagttcc | 23 - 43 |
| LASV08-04 L2R II | taacactgtctacagctatagagc | 2027 - 2050 |
| LASV08-04 L9xF | tgaaacaagtgagagtgcc | 1731 - 1749 |
| LASV08-04 L9xR | gattatcatcaacattcaccagagg | 2338 - 2362 |
| LASV08-04 L3F | ttatgcacatatagtcccatagaagg | 2048 - 2073 |
| LASV08-04 L4R | acatgatagaagactttgatcgag | 4202 - 4225 |
| LASV08-04 L5F | tataggacaagaattcattggc | 4095 - 4116 |
| LASV08-04 L6R | aagactgacaaagtaaagttattggag | 6324 - 6350 |
| LASV08-04 L7F | ttcgatactttcatcatgagtgc | 6270 - 6292 |
| LASV08-04 L8R II | tttacttagagtgtcactaactgacg | 7180 - 7205 |

¹All positions correspond to Nig08-04 (GU481068/GU481069) except for 36E2.

²Dembey, A.H.,et al., Early diagnosis of Lassa fever by reverse transcription-PCR. J Clin Microbiol, 1994.32(12):p.2898-903.
