# Supplementary material for: Genetic characterization of Lassa virus strains isolated from 2012 to 2016 in southeastern Nigeria
Source: PLoS Negl Trop Dis. 2018 Nov 30;12(11):e0006971. doi: 10.1371/journal.pntd.0006971 (PMC6267959; doi:10.1371/journal.pntd.0006971)
Supplement: S2 Table — (DOCX) [file pntd.0006971.s002.docx]

**S2 Table. Sequencing primers for L segment.**

| Primers | Sequences (5′→3′) | Position^1^ |
| --- | --- | --- |
| LASV1seq | ttcaacaaaccaagctgacc | 1023 - 1042 |
| LASV2seq | atcaaaagtcaggatgctagc | 1206 - 1226 |
| LASV1xseq | gattcaagttcatcttagccg | 1346 - 1366 |
| LASV2xseq | tacgccatccattcaagac | 810 - 828 |
| LASV3seq II | aatcgtgatttgaactctgc | 2962 - 2981 |
| LASV4seq II | caactccaactttcaaactgg | 3283 - 3303 |
| LASV3xseq | acttactgtgatccatgct | 3542 - 3560 |
| LASV4xseq | caaattgagagattggttcctg | 2660 - 2681 |
| LASV5seq | atcatactgacaacctcctc | 5044 - 5063 |
| LASV6seq | aacattaggtattttgtgatggc | 5182 - 5204 |
| LASV5xseq | tttgatagctcatagtaggtgtg | 5702 - 5724 |
| LASV6xseq | tgtctagttttaacaatggctc | 4735 - 4756 |
| LASV7seq | ttgtaaaaagttgtcctaccgtc | 6682 - 6705 |
| LASV8seq | acaactttttacaacgagcag | 6675 - 6695 |

^1^All positions correspond to Nig08-04 (GU481069)
