# Supplementary material for: Genetic characterization of Lassa virus strains isolated from 2012 to 2016 in southeastern Nigeria
Source: PLoS Negl Trop Dis. 2018 Nov 30;12(11):e0006971. doi: 10.1371/journal.pntd.0006971 (PMC6267959; doi:10.1371/journal.pntd.0006971)
Supplement: S3 Table — (DOCX) [file pntd.0006971.s003.docx]

**S3 Table. Strains isolated in this study.**

|  |  |  | Accession number | |
| --- | --- | --- | --- | --- |
| Strain | Year | Origin | S segment | L segment |
| Nig12-04 | 2012 | Abakaliki, Nigeria | LC388575 |  |
| Nig12-05 | 2012 | Abakaliki, Nigeria | LC388576 |  |
| Nig12-17 | 2012 | Abakaliki, Nigeria | LC387468 | LC387469 |
| Nig12-27 | 2012 | Abakaliki, Nigeria | LC388577 |  |
| Nig13-03 | 2013 | Abakaliki, Nigeria | LC388578 |  |
| Nig13-04 | 2013 | Abakaliki, Nigeria | LC387470 | LC387471 |
| Nig13-08 | 2013 | Abakaliki, Nigeria | LC388579 |  |
| Nig14-01 | 2014 | Abakaliki, Nigeria | LC388580 |  |
| Nig14-03 | 2014 | Abakaliki, Nigeria | LC387472 | LC387473 |
| Nig14-04 | 2014 | Abakaliki, Nigeria | LC387474 | LC387475 |
| Nig14-06 | 2014 | Abakaliki, Nigeria | LC387476 | LC387477 |
| Nig14-07 | 2014 | Abakaliki, Nigeria | LC388581 |  |
| Nig14-08 | 2014 | Abakaliki, Nigeria | LC388582 |  |
| Nig14-09 | 2014 | Abakaliki, Nigeria | LC387478 | LC387479 |
| Nig14-14 | 2014 | Abakaliki, Nigeria | LC388583 |  |
| Nig14-15 | 2014 | Abakaliki, Nigeria | LC388584 |  |
| Nig14-19 | 2014 | Abakaliki, Nigeria | LC387480 | LC387481 |
| Nig14-20 | 2014 | Abakaliki, Nigeria | LC388585 |  |
| Nig14-22 | 2014 | Abakaliki, Nigeria | LC388586 |  |
| Nig14-24 | 2014 | Abakaliki, Nigeria | LC388587 |  |
| Nig14-25 | 2014 | Abakaliki, Nigeria | LC388588 |  |
| Nig14-40 | 2014 | Abakaliki, Nigeria | LC388589 |  |
| Nig14-41 | 2014 | Enugu, Nigeria | LC388590 |  |
| Nig16-02 | 2016 | Abakaliki, Nigeria | LC387482 | LC387483 |
| Nig16-07 | 2016 | Abakaliki, Nigeria | LC388591 |  |
| Nig16-10 | 2016 | Abakaliki, Nigeria | LC388592 |  |
| Nig16-11 | 2016 | Abakaliki, Nigeria | LC387484 | LC387485 |
| Nig16-13 | 2016 | Abakaliki, Nigeria | LC387486 | LC387487 |
| Nig16-14 | 2016 | Abakaliki, Nigeria | LC387488 | LC387489 |
